# Current and emerging treatment strategies for children with progressive chiasmatic-hypothalamic glioma diagnosed as infants: a web-based survey

**DOI:** 10.1007/s11060-017-2630-6

**Published:** 2017-10-25

**Authors:** Amedeo A. Azizi, Antoinette Y. N. Schouten-van Meeteren

**Affiliations:** 1 Division of Neonatology, Pediatric Intensive Care and Neuropediatrics, Department of Pediatrics and Adolescent Medicine, Medical University of Vienna, Spitalgasse 23, 1090 Vienna, Austria; 2 Department of Pediatric Oncology, Emma Children’s Hospital, Academic Medical Center, Amsterdam, The Netherlands

**Keywords:** Low-grade glioma, LGG, Optic pathway glioma, Chiasmatic hypothalamic glioma, iCHG, CHG, Infants, Children

## Abstract

Treatment of infant hypothalamic chiasmatic glioma (iCHG) is challenging, about 30% of the children progress during chemotherapy. Despite subsequent treatments the 5 year overall-survival rate is only 70%. This study investigates treatment strategies currently applied for progressive iCHG. A web-based questionnaire was sent out to the members of the SIOPE Brain Tumour Group asking for current second and third line strategies at progression *during* and *after the end* of first line therapy. The questionnaire was answered by 47 paediatric oncologists from 15 countries. iCHG progressing *during* first line therapy with carboplatin-vincristine would be considered for treatment with alternative chemotherapy by 17 (36%) and with surgery plus chemotherapy by 27 respondents (58%). Components suggested for second line were vinblastine (62%), cisplatin (34%) and cyclophosphamide (26%). For third line therapy bevacizumab (BVZ) was considered as suitable by respondents in 53% (often with irinotecan 40%) and vinblastine by 34% respectively. Experience with BVZ in CHG is shown by 53% of respondents regarding at least 95 patients (median treated 1–5 patients per respondent at any age) with a median BVZ administration over 12 months. Effectiveness was reported varying between stable disease and regression while complications were rarely stated (proteinuria, hypertension, bleeding). BVZ would be available to 85% of respondents as therapeutic option for iCHG patients. Multiple anti-neoplastic drug regimens are applied for progressive iCHG, partly considered in combination with surgery if safely feasible. BVZ is commonly used at a satisfactory level in third line, mainly combined with irinotecan.

**Electronic supplementary material:**

The online version of this article (doi:10.1007/s11060-017-2630-6) contains supplementary material, which is available to authorized users.

## Background

Low-grade gliomas (LGG) are the most common brain tumours in childhood, accounting for approximately 35% during the first year of life and up to 50% in older children [1]. The chiasmatic/hypothalamic region is the predilective location in infants, and 20% of all intracranial tumours in children below the age of 2 years occur in this region [2]. Overall, infant chiasmatic hypothalamic glioma (iCHG) still constitutes a rare entity [3]. Young age has been demonstrated to represent a risk factor for progression in children with LGG, translating into an inferior overall survival rate [4–6]. Whereas surgical resection improves outcome in LGG outside of the chiasmatic/hypothalamic region [4, 5], it does not in children with iCHG and may cause additional harm such as infarction or impaired neurological and endocrine function [7, 8]. The vulnerability of the very young brain hampers the application of radiotherapy at this age [9–11]. Systemic chemotherapy is required and the regimen most commonly used in first-line comprises carboplatin and vincristine in different time-schedules and dosing [5, 12]. Treatment with carboplatin and vincristine over a period of 18 months represents the current strategy proposed by the Societé Internationale d’Oncologie Pédiatrique in Europe (SIOPE) [13]. iCHG is characterised by multiple sequential progressions and the necessity to apply subsequent treatment cycles [3, 6, 14, 15]. Multiple different therapy regimens have been reported for progressive LGG, including monotherapies with vinblastine [16], temozolomide [17], or carboplatin [18, 19] as well as chemotherapy combinations such as TPCV (thioguanine, procarbazine, CCNU (1-(2-chloroethyl)-3-cyclohexyl-1-nitrosourea) and vincristine) [15] or repeated carboplatin/vincristine [15]. These relapse strategies were shown to efficiently influence survival.

The present survey was conducted following intense discussions among the SIOPE LGG working group on possible treatment strategies for infants with progressive CHG. The survey therefore aimed at evaluating treatment strategies currently adopted for progressive iCHG, i.e. progressive tumour in children diagnosed during the first year of life. Following primary reports on the efficacy of bevacizumab (BVZ) in patients with LGG [20–23], and the inefficacy of conventional chemotherapy in approximately 30% of iCHG patients, the use of BVZ was discussed as possible treatment strategy. The working group recognised the lack of data for the use of BVZ in such a vulnerable young patient cohort, necessitating a detailed evaluation of its use in iCHG patients. The current professional experiences in application of BVZ in paediatric CHG were therefore investigated in more detail.

## Methods

A web-based questionnaire on experiences and preferences on relapse treatment for iCHG (during or following first line treatment with carboplatin/vincristine) was created at http://www.thesistools.de (see supplementary material). Per definition, tumours were considered as infant CHG if diagnosed during the first year of life. Treatment at progression or at relapse after the end of intial therapy (independent of the age at relapse) was addressed in the present survey. The survey took around 15 min to fill in and consisted of questions regarding treatment strategies, drugs applied for systemic therapy and experience with bevacizumab in CHG patients. Additional questions were asked on characteristics of the participating medical specialist: profession, country of origin, years of clinical experience, yearly amount of children treated with a brain tumour and the subgroups of LGG, CHG as well as iCHG specifically. As the survey aimed at assessing general treatment strategies, it did not collect data on patient characteristics (e.g. age at relapse, pattern of relapse or initial symptoms such as diencephalic syndrome).

The invitation to participate in the survey with a directly accessible link was sent to specialists in the field of paediatric neuro-oncology consisting of the 220 members of the multidisciplinary SIOPE Brain Tumour Group via email in February 2014 and repeated after 3 weeks.

## Results

### Respondents

The questionnaire was answered by 47 paediatric oncologists from 15 countries (Austria, Canada, Czech Republic, Denmark, France, Germany, Italy, Netherlands, Poland, Portugal, Slovakia, Spain, Sweden, United Kingdom, United States). Two neuro-surgeons were omitted from the analysis as they indicated not to be involved in the decision on systemic treatment approaches. The professional experience of the respondents was reflected by the fact that 85% worked in a clinic with > 20 new brain tumour patients per year. Their years of experience in neuro-oncology were as follows: <5 years n = 5, 5–10 years n = 8, 10–20 years n = 18, > 20 years n = 15 and missing n = 1. 81% treated 1–6 patients with newly diagnosed CHG yearly, 19% seven or more patients per year. The reported rate of iCHG was low, during a 5 year period 57% of respondents cared for 1–3, 23% for 4–6, while 20% for more than six patients with iCHG.

### Duration of first line treatment

Considering the high rate of patients progressing after chemotherapy, the survey asked on the opinion of prolonging first line chemotherapy (see Table 1). The majority of respondents (81%) administered first line treatment over a period of 18 months, a third (36%) indicated to be willing to consider prolonging first line treatment. Vinblastine was the most prevalent suggestion for such a prolongation.

**Table 1:** Strategies for progression of infant chiasmatic hypothalamic glioma

Duration of 1st line treatment		n	%
Length of primary treatment	12 months	7	15
	18 months	38	81
	60 weeks	1	2
	Prolonged duration	1	2
Considering prolonged maintenance after firstline therapy	Yes	17	36
	No	30	64

Types of treatment considered	2nd line treatment at progression *during* carboplatin/vincristine		Progression *after* end of carboplatin/vincristine		3rd line treatment if not responsive or early progression	
	n	%	n	%	n	%
Surgery only	–		–	–	3	6
Surgery and continue/restart carboplatin/vincristine	4	9	3	6	3	6
Surgery and other chemotherapy	23	49	15	32	20	43
Different chemotherapy	17	36	9	19	3	6
Restart carboplatin/vincristine			13	27	–	
Monotherapy targeted	–		–		4	9
Targeted and other chemo	1	2	2	4	14	30
Brachytherapy	–		–		–	
External beam radiotherapy	2	4	–		–	
Missing			1	2		
Total	47	100	47	100	47	100

Drugs applied (multiple answers allowed)						
Median Range	2 1–6		2 1–8		2 1–7	
Number of respondents Missing	47		46 1		47	
Actinomycin D	2	4	3	6	3	6
Bevacizumab	9	20	5	11	25	53
Carboplatin	4	9	10	22	2	4
CCNU	1	2	1	2	5	11
Cisplatin	16	34	10	22	3	6
Cyclophosphamide	12	26	9	20	2	4
Etoposide	10	21	8	17	2	4
Everolimus	–		–		3	6
Ifosfamide	1	2	1	2	–	–
Imatinib mesylate	–		1	2	4	9
Irinotecan	5	11	3	6	19	40
MEK inhibitor	–		1	2	1	
Methotrexate	1	2				
Nilotinib	5	11	5	11	3	6
Procarbazine	2	4	2	4	6	13
Temozolomide	2	4	4	9	2	4
Thioguanine	2	4	2	4	5	11
Vinblastine	29	62	25	54	16	34
Vincristine	11	23	16	35	9	19
Other drugs			2^a^	4	8^b^	19

^a^Vinorelbine

^b^Navelbine, fluvastatine, dacarbazine, thalidomide, celecoxib, fenofibrate

### Treatment strategies at progression

Therapeutic strategies adopted and systemic therapies administered at progression in patients with iCHG are summarised in Table 1. Progressive disease *during* first line therapy with carboplatin - vincristine would be treated with different chemotherapy by 17 (36%) and with surgery plus chemotherapy by 27 respondents (58%). Vinblastine was selected as single chemotherapeutic agent in 62% followed by cisplatin (34%) and cyclophosphamide (26%) in multi-agent combinations.

In case of progression *after the end* of combined carboplatin—vincristine chemotherapy 54% of respondents would favour vinblastine monotherapy while about 44% would choose a platinum derivative, 35% vincristine and 20% cyclophosphamide, mostly in combination therapy. In addition to systemic therapy 38% of respondents would consider a neurosurgical option (if safely feasible) in combination with further chemotherapy.

In third line respondents would use BVZ in 53%, in 19/25 (76%) combined with irinotecan. Vinblastine is considered by 34%.

The actual experiences in application of BVZ are listed in Table 2. Bevacizumab was applied in at least 95 patients with CHG by 53% of respondents, mostly combined with irinotecan. Median duration of BVZ therapy was 12 months (range 4–24). An effect was reported for all patients with at least stable disease while severe complications were rarely mentioned (proteinuria, hypertension and bleeding, each indicated by a single respondent). Although BVZ is not licensed for use in LGG, 85% of respondents stated that BVZ would be available at their centre for use in patients with progressive iCHG.

**Table 2:** Application of bevacizumab (BVZ) in patients with (infant) chiasmatic hypothalamic glioma (n = number of respondents)

	n	%
**Experience with BVZ in CHG**		
Yes	25	53
No	22	47
**Experience of BVZ in CHG patients per respondent**		
None	22	47
1	3	6
2–5	16	34
6–10	2	4
11–15	3	6
> 15	1	2
**% of CHG patients with stable disease or regression (n = number of respondents, not patients treated!)**		
0–20%	1	4
20–40%	4	16
40–60%	5	20
> 60%	15	60
Not applicable	22	
**Patients with CHG treated with BVZ below 1 year of age**		
None	30	64
1	11	23
2–5	5	11
6–10	–	
11–15	–	
> 15	–	
Missing	1	2
**Duration of bevacizumab treatment (in months)**		
Not available	25	
0–3	–	
4–6	3	
7–9	3	
10–12	11	
13–18	1	
19–24	4	
> 24	–	
**Major complications**		
Yes	3	12
No	22	88
**Type of complications**		
Intracranial bleeding	0	
Bleeding outside of CNS	1	
Hypertension	1	
Proteinuria	1	
Wound healing problems	0	
Other	0	
**Combination of BVZ with other drugs**		
Yes	23	92
No	2	8
**Drugs applied together with BVZ**		
Irinotecan	20	
Vinblastine	1	
Thalidomide	1	
Missing	1	

### Other findings

Radiotherapy was not considered an option in second and third line therapy (see Table 1). Respondents indicated that the median age at which they would consider starting external beam radiotherapy was 7 years (range 1–18, “never” n = 2). Reasons to start radiotherapy were collected as open answers and are therefore challenging to evaluate. Forty-one out of 45 evaluable respondents would only consider radiotherapy if the tumour was chemo-irresponsive (most indicating failure to more than two to three lines of systemic therapy).

## Discussion

iCHG has a high risk of progression (already occurring during chemotherapy in 70% of patients) and exhibits a poor overall survival of 70% [3–6]. Due to the very low incidence of iCHG prospective studies specifically targeting the infant population are difficult to imply and data on treatment efficacy are scarce [3]. Consequently there are no clear guidelines for relapse therapy and so far no drug choice has shown superior effectiveness. Opinions from experts in the field are highly relevant to increase insight in current practice. As patients with iCHG suffer multiple relapses [14] the present survey was conducted among the SIOPE Brain Tumour Group to identify presently applied treatment strategies, specifically the choices made in application of chemotherapeutic drugs. By inviting all members of the SIOPE Brain Tumour Group the present survey was reaching the broad SIOPE community of paediatric neuro-oncologists, reflected by respondents from 15 countries.

According to the current recommendations of the SIOPE LGG group, carboplatin and vincristine are mostly administered over a period of 18 months in first line therapy, but a third of responding experts would consider prolongation of first line treatment to ameliorate PFS.

The present survey underlines that systemic therapies are considered the mainstay of relapse therapy in iCHG. Even if neurosurgery is (re-)considered, subsequent systemic therapy approaches are deemed necessary to sustain a possible tumour reduction achieved by neurosurgery. Accordingly none of the respondents would select a stand-alone neurosurgical approach at first tumour progression and 6% at second progression.

Radiotherapy was not considered an option in second and third line therapy by respondents of this survey. The vast majority would even only consider radiotherapy after failure of multiple lines of systemic therapy. This reasoning will probably be based on the possible devastating side effects of cranial radiotherapy in small children [10, 11]. Also radiotherapy has to be considered a major risk factor for delayed mortality in adult survivors of childhood low-grade glioma [9].

The survey revealed that a wide variety of anti-neoplastic agents are applied in subsequent therapy lines.

At first progression vinblastine is chosen most often as 2nd line treatment, 62% for progression during carboplatin-vincristine and 54% for progression after the end first line treatment. The excellent tolerability and efficacy of vinblastine in LGG has been documented earlier and its efficacy was also reported in young children with iCHG and those with diencephalic syndrome, either alone or in combination with carboplatin [16, 24, 25]. Vinblastine was recently even reported as maintenance after BVZ-irinotecan therapy [26].

A wide variety of drugs is used in case of progression during and/or after first line chemotherapy. We could not define specific subgroups of professionals for instance by nationality and/or years of experience, who provided specific alternative chemotherapy regimens. Only the application of cisplatin in combination with etoposide was reported by Italian neuro-oncologists as published by Massimino et al. [27, 28] Although the decreased dose diminished ototoxicity [28], it still seems potentially ototoxic which is especially relevant in children with impaired vision where dual sensory loss is risked. The use of more intense chemotherapy regimens might reflect the fact that children with iCHG progressing under chemotherapy exhibit an increased mortality. The data also mirror the advice in the earlier LGG 2004 protocol to start cyclophosphamide and cisplatin alternating in cycles in parallel to vincristine in case of carboplatin allergy. The choices for actinomycin and methotrexate are scarce, although relevance has been shown in older literature reports [29–31].

The antiangiogenic agent BVZ, a monoclonal antibody targeting vascular endothelial growth factor is approved for oncologic treatment in adults and was consecutively applied in a variety of paediatric CNS malignancies. In childhood LGG several studies demonstrated benefit of BVZ, specifically in patients with CHG. Packer et al. were the first to report clinical and radiologic responses in ten cases with multiple relapsed LGG with a combination of two-weekly BVZ and irinotecan [20]. Kalra et al. showed similar results in 16 children, including 8 with disseminated disease [32]. It was demonstrated that a majority of patients relapsed after stopping therapy, but re-introduction of BVZ was able to re-induce response and maintained responses were seen after prolonging intervals to 3 weeks [23]. Besides tumour control, improvement of visual function was reported in eight out of nine patients (four stable vision, four improved vision) with previous visual loss from both studies [22, 32]. The toxicity profile of BVZ in children seems acceptable with temporary proteinuria, hypertension, impaired wound healing and rarely bleeding or osteonecrosis [23, 33–36].

Indeed application of BVZ for progressive iCHG seems to be a hopeful strategy as shown in the experiences of 53% of our respondents reflecting experience in at least 95 patients. The previously published results of BVZ in LGG incited us to investigate the current practice of BVZ application in progressive CHG in the clinical practice of paediatric neuro-oncology. The results of the survey suggest that while vinblastine is widely applied in second line, BVZ was indicated as most prevalent choice in third line therapy with a majority of clinical responses despite multiple progressions of their LGG.

BVZ is not approved for the use in children with LGG and expensive compared to conventional chemotherapy. Considering its effectiveness in LGG [20, 21] and the possible functional benefit of visual improvement in children with iCHG [22, 23], its use may still be considered. Results from an ongoing randomised phase 2 trial on BVZ and vinblastine in chemotherapy-naïve children with LGG (NCT02840409) are eagerly awaited.

Following the increasing understanding of molecular pathways involved in LGG pathogenesis [37], a novel treatment strategy targeting at the MAPK pathway was evaluated. However, despite early hope, RAF inhibitor sorafenib revealed not only ineffective, but proved even detrimental due to an activating feed-back loop causing rapid progression of recurrent LGG [38]. MEK inhibitors block signalling of the MAPK pathway downstream of RAF, thereby thought to circumvent the problems encountered in first generation BRAF inhibitors. Primary clinical data have been presented on the efficacy of MEK inhibitors in relapsed LGG, and results from phase 1 and 2 trials in the paediatric population are emerging [39, 40].

Since a majority of patients with LGG become long term survivors, more insight is needed in the late outcome by gathering data in a common registry [41]. This is specifically true for assessing long term sequelae resulting in children with iCHG caused by the tumour itself or its subsequent treatment. We therefore advocate a registry for all children with multiple subsequent treatment strategies at progression of CHG.

### Strengths and limitations

Our survey provides insights in the opinions of a broad group of experienced paediatric neuro-oncologists and reflects clinical practice from many different countries in the choices made in treatment of progressive iCHG. The information regarding BVZ in clinical practice refers to at least 95 patients, extending the information on the use of BVZ, so far limited to reports published on 25 CHG patients treated with BVZ. Due to the nature of an online survey there was the possibility that multiple respondents from one treatment centre may have answered the questionnaire. Indeed, two IP-addresses were used by two respondents each. All four could be identified as independent respondents; one of them was neurosurgeon and left out from analysis (see above). Only 47/220 persons invited via email have responded to the survey, maybe distorting the results. As the mailing list of SIOPE also included neurologists, radiotherapists, neurosurgeons, neuro-pathologists, psychologists, endocrinologists, statisticians, basic scientists and ophthalmologists who were not addressed by the present survey, the percentage of paediatric neuro-oncologists responding is much higher than suggested by this number.

The efficacy of other targeted drugs, such as specification of MEK inhibitors were not implicated in our survey yet. The survey allowed however to name alternative drugs and the application of a MEK inhibitor was indicated only twice by the respondents. The group of MEK inhibitors deserves specific attention in a possible follow-up survey in the future.

Although clinical response to BVZ is reported by the majority of respondents our survey did not extrapolate on the responses in vision and the differences between children with or without neurofibromatosis type 1.

## Conclusion

Multiple different drug regimens are applied for progressive iCHG. Also in case of a neurosurgical approach subsequent anti-neoplastic treatment is widely applied. The application of targeted drugs emerges as novel treatment strategy. While vinblastine is the most common drug used in second line treatment, BVZ is often used in third line, mostly in combination with irinotecan. Prospective data on the efficacy of BVZ in LGG are needed and should include the infant population. Whereas the present findings cannot replace the lack of prospective trials for this small group of patients with progressive iCHG, they clearly reflect current strategies applied by an international community of paediatric neuro-oncologists. Still, treatment decisions have to be made on a patient per patient basis by an experienced interdisciplinary paediatric neuro-oncology team as depicted by the wide variety of possible therapeutic strategies. We advocate an international registry for children with (i)CHG to prospectively gather information on the multiple progressions and subsequent treatment strategies.

## Electronic supplementary material

Below is the link to the electronic supplementary material.

Supplementary material 1 (PDF 1278 KB)

## Abbreviations

BVZ: Bevacizumab
CHG: Chiasmatic hypothalamic glioma
iCHG: Infant chiasmatic hypothalamic glioma
LGG: Low-grade glioma
SIOPE: Societé Internationale d’Oncologie Pédiatrique in Europe
TPCV: Thioguanine, procarbazine, CCNU (1-(2-chloroethyl)-3-cyclohexyl-1-nitrosourea) and vincristine
VEGF: Vascular endothelial growth factor
