# Supplementary material for: Current and emerging treatment strategies for children with progressive chiasmatic-hypothalamic glioma diagnosed as infants: a web-based survey
Source: J Neurooncol. 2017 Oct 25;136(1):127–34. doi: 10.1007/s11060-017-2630-6 (PMC5754463; doi:10.1007/s11060-017-2630-6)
Supplement: Supplementary file 1 — Supplementary material 1 (PDF 1278 KB) [file 11060_2017_2630_MOESM1_ESM.pdf]

STEP 4. Preview

Tip: Click on a text to edit.

Add an element

Seite: 1

Fortschritt_Basis | Fortgang | kopiëren | anpassen

[Fortgangsbalken]

11%

TREATMENT_OF_PROGRESSIVE_OPG_<1_yr | Überschrift | kopiëren | anpassen

Treatment of Progressive Optic Pathway Glioma in Infants

Einleitung_[KLICKEN,_UM_ANZUPASSEN] | Zwischentext | kopiëren | anpassen

Sporadic infant chiasmatic / hypothalamic glioma (ICHG) has to be considered as a chronic disease and almost invariably will necessitate subsequent treatment episodes at progression (i.e. second-line or third-line treatment).

The dismal prognosis of ICHG made us design a specific questionnaire for this disease. It shall help to characterise current treatment decisions for progressive infant chiasmatic / hypothalamic glioma (ICHG), i.e. children diagnosed during the first year of life.

All questions below therefore ONLY concern INFANTS with chiasmatic / hypothalamic glioma (i.e. DIAGNOSED during the first year of life).

authors | Zwischentext | kopiëren | anpassen

This survey has been initiated by Amedeo A. Azizi and A.Y.N. Schouten - van Meeteren on behalf of the

SIOP-E Low Grade Glioma working group.

Neue Seite | kopiëren | anpassen

Next

Seite: 2 | PAGE_2

previous

22%

LIST_of_QUESTIONS | Static Text | copy | adjust

QUESTIONS to following topics will be asked.

- Progression DURING Carboplatin / VINCRISTIN
- Progression DURING Carboplatin / VINBLASTIN
- Progression AFTER Therapy
- LENGTH of 1st line treatment
- BEVACIZUMAB
- RADIATION THERAPY
- GENERAL INFORMATIONS

Answering the questionnaire will take 10-15 minutes.
Thank you for your time.

New page | copy | adjust

Next

Page: 3 | PROGRESSION_DURING_Carbo/VCR

previous

33%

reminder_patient_cohort | Title | copy | adjust

All questions below ONLY concern children with Chiasmatic Hypothalamic Glioma (ICHG) DIAGNOSED during the FIRST year of life.

PROGRESSION_DURING_CARBOPLATIN_/_V | Title | copy | adjust

Progression DURING Carboplatin / VINCRISTINE

treatment_progr._DURING_Carbo_VCR | Multiple Choice Max. 1 | copy | adjust

1

How do you treat a child progressing during carboplatin / VINCRISTINE chemotherapy?

- ◯ (Intensified) CTX (see selection below)
- ◯ Targeted monotherapy (e.g. bevacizumab, imatinib) (see selection below)
- ◯ Targeted agent like bevacizumab combined with other drug(s)
- ◯ Surgery (if safely feasible)
- ◯ Surgery (if safely feasible) and continuation of LGG chemotherapy
- ◯ Surgery (if safely feasible) and other CTX (see selection below)
- ◯ Brachytherapy
- ◯ External beam radiotherapy
- ◯ Other (specify?) ____
- ◯ Remarks ____

drugs_in_second_line_(DURING) | Multiple Choice | copy | adjust

2

Which drug(s) do you actually use in second line (drugs listed below identified by literature review)?

(multiple selections are possible in case you apply a multiple-drug regime)

- ☐ Actinomycin
- ☐ Bevacizumab (Avastin)
- ☐ Carboplatin
- ☐ CCNU
- ☐ Cisplatin
- ☐ Cyclophosfamide
- ☐ Etoposide
- ☐ Everolimus
- ☐ Ifosfamide
- ☐ Imatinib mesylate (Gleevec)
- ☐ Irinotecan
- ☐ Nilotinib
- ☐ Procarbazine
- ☐ Temozolomide
- ☐ Thioguanine
- ☐ Vinblastin
- ☐ Vincristin
- ☐ Other (specify?) ____

☐ Remarks

3rd_line_progr._DURING_Carbo_VCR | Multiple Choice Max. 1 | copy | adjust

3

What do you consider as third line therapy if not responsive or early progressive ?

- (Intensified) CTX (see selection below)
- Targeted monotherapy (e.g. bevacizumab, imatinib) (see selection below)
- Targeted agent like bevacizumab combined with other drug(s)
- Surgery (if safely feasible)
- Surgery (if safely feasible) and continuation of LGG chemotherapy
- Surgery (if safely feasible) and other CTX (see selection below)
- Brachytherapy
- External beam radiotherapy
- Other (specify?)
- Remarks

Drugs_third_line_(early_progr.) | Multiple Choice | copy | adjust

4

Which drug(s) do you use in third line (drugs listed below identified by literature review)?

(multiple selections are possible in case you apply amultiple-drug regime)

☐ Actinomycin
☐ Bevacizumab (Avastin)
☐ Carboplatin
☐ CCNU
☐ Cisplatin
☐ Cyclophosfamide
☐ Etoposide
☐ Everolimus
☐ Ifosfamide
☐ Imatinib mesylate (Gleevec)
☐ Irinotecan
☐ Nilotinib
☐ Procarbazine
☐ Temozolomide
☐ Thioguanine
☐ Vinblastin
☐ Vincristin
☐ Other (specify?)

☐ Remarks

New page | copy | adjust

Next

Page: 4 | progression_DURING_Carbo_VBL

previous

44%

reminder_patient_cohort | Title | copy | adjust

All questions below ONLY concern children with Chiasmatic Hypothalamic Glioma (ICHG) DIAGNOSED during the FIRST year of life.

PROGR_DURING_CARBO_/_VBL | Title | copy | adjust

Progression DURING Carboplatin / VINBLASTIN

treatment_progr._DURING_Carbo_VBL | Multiple Choice Max. 1 | copy | adjust

5

How do you treat a child progressing during carboplatin / VINBLASTIN chemotherapy?

- (Intensified) CTX (see selection below)
- Targeted monotherapy (e.g. bevacizumab, imatinib) (see selection below)
- Targeted agent like bevacizumab combined with other drug(s)
- Surgery (if safely feasible)
- Surgery (if safely feasible) and continuation of LGG chemotherapy
- Surgery (if safely feasible) and other CTX (see selection below)
- Brachytherapy
- External beam radiotherapy
- Other (specify?)
- Remarks

drugs_in_second_line_(DURING)_VBL | Multiple Choice | copy | adjust

6

Which drug(s) do you actually use in second line (drugs listed below identified by

literature review)?

(multiple selections are possible in case you apply a multiple-drug regime)

- Actinomycin
- Bevacizumab (Avastin)
- Carboplatin
- CCNU
- Cisplatin
- Cyclophosfamide
- Etoposide
- Everolimus
- Ifosfamide
- Imatinib mesylate (Gleevec)
- Irinotecan
- Nilotinib
- Procarbazine
- Temozolomide
- Thioguanine
- Vinblastin
- Vincristin
- Other (specify?)
- Remarks

3rd_line_progr._DURING_Carbo_VBL | Multiple Choice Max. 1 | copy | adjust

7

What do you consider as third line therapy if not responsive or early progressive ?

- (Intensified) CTX (see selection below)
- Targeted monotherapy (e.g. bevacizumab, imatinib) (see selection below)
- Targeted agent like bevacizumab combined with other drug(s)
- Surgery (if safely feasible)
- Surgery (if safely feasible) and continuation of LGG chemotherapy
- Surgery (if safely feasible) and other CTX (see selection below)
- Brachytherapy
- External beam radiotherapy
- Other (specify?)
- Remarks

Drugs_third_line_(early_progr.)_VBL | Multiple Choice | copy | adjust

8

Which drug(s) do you use in third line (drugs listed below identified by literature review)?

(multiple selections are possible in case you apply amultiple-drug regime)

- Actinomycin
- Bevacizumab (Avastin)
- Carboplatin
- CCNU
- Cisplatin
- Cyclophosfamide
- Etoposide
- Everolimus
- Ifosfamide
- Imatinib mesylate (Gleevec)
- Nilotinib

Irinotecan

- [ ] Procarbazine
- [ ] Vincristin
- [ ] Temozolomide
- [ ] Thioguanine
- [ ] Vinblastin
- [ ] Other (specify?)
- [ ] Remarks

New page | copy | adjust

NEXT

Page: 5 | Progression_AFTER_therapy

previous

56%

reminder_patient_cohort | Title | copy | adjust

All questions below ONLY concern children with Chiasmatic Hypothalamic Glioma (ICHG) DIAGNOSED during the FIRST year of life.

Title_Progression_after_ther | Title | copy | adjust

Progression AFTER therapy

Treatment_AFTER_end_1st_line_CTX | Multiple Choice Max. 1 | copy | adjust

9

How do you treat a child progressing AFTER THE END of primary chemotherapy

- ( ) Rechallenging to carboplatin / vincristin or carboplatin / vinblastin
- ( ) Other CTX (see selection below)
- ( ) Targeted monotherapy (e.g. bevacizumab, imatinib) (see selection below)
- ( ) Targeted agent like bevacizumab combined with other drug(s)

- Surgery (if safely feasible)
- Surgery (if safely feasible) and continuation of LGG chemotherapy
- Surgery (if safely feasible) and other CTX (see selection below)
- Brachytherapy
- External beam radiotherapy
- Other (specify?)
- Remarks

Drugs_progr_after_end_1st_line | Multiple Choice | copy | adjust

10

Which drug(s) do you use in second line (drugs listed below identified by literature review)?

(multiple selections are possible in case you apply amultiple-drug regime)

- Actinomycin
- Bevacizumab (Avastin)
- Carboplatin
- CCNU
- Cisplatin
- Cyclophosfamide
- Etoposide
- Everolimus
- Ifosfamide
- Imatinib mesylate (Gleevec)
- Irinotecan
- Nilotinib
- Procarbazine
- Vincristin
- Temozolomide
- Thioguanine
- Vinblastin
- Other (specify?)
- Remarks

New page | copy | adjust

Next

Page: 6 | LENGTH_of_treatment

previous

67%

reminder_patient_cohort | Title | copy | adjust

All questions below ONLY concern children with Chiasmatic Hypothalamic Glioma (ICHG) DIAGNOSED during the FIRST year of life.

LENGTH_of_treatment | Title | copy | adjust

LENGTH of first line treatment

LENGTH_of_treatment | Multiple Choice Max. 1 | copy | adjust

11

How long do you treat in first line?

- 12 months
- 18 months
- other

duration_1st_line_Carbo_VCR | Drop down | copy | adjust

12

In case of carboplatin / vincristine (e.g. SIOP LGG 2004): do you presently stop after 18 months or add a prolonged maintenance?

:: Please choose ::

contiuring_Carbo_VCR_prolongued | Drop down | copy | adjust

13

If you prolong 1st line Carbo / VCR, do you add more Carbo / VCR cycles?

:: Please choose ::

Drugs_prolonged_maintenance_actual | Multiple Choice | copy | adjust

14

If you give other drugs, please specify

(multiple selections are possible in case you apply amultiple-drug regime)

- Actinomycin
-
- Carboplatin

Bevacizumab (Avastin)

- [ ] CCNU
- [ ] Cisplatin
- [ ] Cyclophosfamide
- [ ] Etoposide
- [ ] Everolimus
- [ ] Ifosfamide
- [ ] Imatinib mesylate (Gleevec)
- [ ] Irinotecan
- [ ] Nilotinib
- [ ] Procarbazine
- [ ] Vincristin
- [ ] Temozolomide
- [ ] Thioguanine
- [ ] Vinblastin
- [ ] Other (specify?)
- [ ] Remarks

consider_prolonged_maintenance | Drop down | copy | adjust

15

Would you consider prolonged maintenance of first line therapy?

:: Please choose :: ▼

Drugs_prolonged_maintenance_conside | Multiple Choice | copy | adjust

16

If so: which therapy?

(multiple selections are possible in case you apply amultiple-drug regime)

- [ ] Actinomycin
- [ ] Bevacizumab (Avastin)
- [ ] Carboplatin
- [ ] CCNU
- [ ] Cisplatin
- [ ] Cyclophosfamide
- [ ] Etoposide
- [ ] Everolimus
- [ ] Ifosfamide
- [ ] Imatinib mesylate (Gleevec)
- [ ] Irinotecan
- [ ] Nilotinib
- [ ] Procarbazine
- [ ] Temozolomide
- [ ] Thioguanine
- [ ] Vinblastin
- [ ] Vincristin
- [ ] Other (specify?)
- [ ] Remarks

New page | copy | adjust

Next

Page: 7 | BVZ

previous

78%

Title_Use_of_BVZ | Title | copy | adjust

Use of Bevacizumab in children with chiasmatic hypothalamic glioma

BVZ_availibility | Drop down | copy | adjust

17

Was bevacizumab available to you in the past for treatment of children with chiasmatic / hypothalamic glioma?

:: Please choose ::

Experience_with_BVZ_in_infant_OPG | Drop down | copy | adjust

18

Do you have experience in the use of BVZ in children with chiasmatic / hypothalamic glioma?

:: Please choose ::

How_many_treated_BVZ | Multiple Choice Max. 1 | copy | adjust

19

If yes, how many were treated at your centre?

- Not applicable
- 1
- 2 to 5
- 6 to 10
- 11 to 15
- more than 15 (how many?)

Percentage_response_BVZ	Multiple Choice Max. 1	copy	adjust

20

If yes, how many responded with stable disease or regression?

- Not applicable
- 0 to 20 %
- 20 to 40 %
- 40 to 60 %
- more than 60 %
- specify if possible

How_many_BVZ_under_1	Multiple Choice Max. 1	copy	adjust

21

If yes, how many were below one year of age?

- Not applicable
- 1
- 2 to 5
- 6 to 10
- 11 to 15
- more than 15 (how many?)

BVZ_duration	Drop down	copy	adjust

22

How long do you usually treat children with CHG using Bevacizumab?

:: Please choose ::

Combination_BVZ	Multiple Choice Max. 1	copy	adjust

23

Was BVZ combined with another drug? if yes, specify.

- No
- Yes

BVZ_complications | Multiple Choice | copy | adjust

24

Where there major complications using BVZ in these children?

- No
- Yes (please specify)

Future_availability_BVZ | Multiple Choice Max. 1 | copy | adjust

25

Would BVZ be available to you in the future for treatment of young children with OPG (outside of a treatment protocol with bevacizumab provided)?

- No
- Yes (please specify)

New page | copy | adjust

Next

Page: 8 | RTX

previous

89%

reminder_patient_cohort | Title | copy | adjust

All questions below ONLY concern children with Chiasmatic Hypothalamic Glioma (ICHG) DIAGNOSED during the FIRST year of life.

title_RTX | Title | copy | adjust

Radiation therapy

title_external_RTX | Title | copy | adjust

External radiation therapy

Age_RTX | Drop down | copy | adjust

26

Above which age (in years) do you consider applying external beam radiotherapy?
(answer in years)

:: Please choose ::

indications_RTX | Multiple Text Lines | copy | adjust

27

What do you consider as indications to apply external beam radiotherapy?

1
2
3
4
5

contraindications_RTX | Multiple Text Lines | copy | adjust

28

What do you consider the most important contra-indications to external beam radiotherapy, i.e. which lead to withholding it?

1
2
3
4
5

title_brachyther | Title | copy | adjust

Brachytherapy

brachytherapy_option_y-n | Drop down | copy | adjust

29

Do you consider brachytherapy as an option?

:: Please choose ::

brachyther_avail | Drop down | copy | adjust

30

Is it available for your patients?

:: Please choose ::

brachyther_indic | Multiple Text Lines | copy | adjust

31

15.2. If Brachytherapy is available: What are the indication to perform brachytherapy?

1

2

3

4

5

New page | copy | adjust

Next

Page: 9 | SEITE_General_issues

previous

100%

TITLE_General_issues | Title | copy | adjust

General issues

country | Multiple Text Lines | copy | adjust

32

Country / Oncologic centre

Country

Oncologic centre (place of work)

brain_tu_pts_per_year | Multiple Choice | copy | adjust

33

Please indicate the number of new brain tumour patients at your centre per year?

- less than 5
- 5 to 10
- 10 to 20
- 20 to 30
- 30 to 40
- more than 40

Nr_patients_OPG_per_yr | Multiple Choice | copy | adjust

34

How many new patients with optic pathway glioma are seen at your centre per year?

- none
- 1 to 3
- 4 to 6
- 7 to 10
- 10 to 15
- more than 15

Nr_infants_OPG_5_yrs | Multiple Choice | copy | adjust

35

How many infants with OPG present in five years

- [ ] less than 1
- [ ] 1
- [ ] 2 to 3
- [ ] 4 to 6
- [ ] 7 to 8
- [ ] more than 8
- [ ] comments

personal_information | Title | copy | adjust

Personal information

profession | Multiple Choice Max. 1 | copy | adjust

36

Profession

- ( ) Paediatric oncologist
- ( ) Neurosurgeon
- ( ) Radiotherapist
- ( ) Other

EXPERIENCE | Drop down | copy | adjust

37

Years of experience

:: Please choose :: ▼

Personal_information | Multiple Text Lines | copy | adjust

38

Please enter personal details below (optional)

Name

email

Open_comments | Essay / text area | copy | adjust

39

If there are issues that have not been raised, please enter your comments:

Submit | Submit button | copy | adjust

Submit

Final text

x | Final Text | copy | adjust

Thank you very much for answering the questionnaire!

Please send us your feedback, comments and remarks

Spam prevention 1 + 4 =

About Us | Privacy | Terms & Conditions

send feedback

14/04/2016 © ThesisTools.de Developed by: Joan van Rixtel Designed by: Marketinge PHP Framework: Symfony2 Thanks to: Coen Dörge, Ulrike Grafberger
